# ANCA associated vasculitis: experience of a tertiary care referral center

**DOI:** 10.1590/2175-8239-JBN-2018-0040

**Published:** 2018-08-09

**Authors:** Rafia Chaudhry, Anum Bilal, Adam Austin, Swati Mehta, Loay Salman, Llewellyn Foulke, Paul Feustel, Roman Zuckerman, Arif Asif

**Affiliations:** 1 Albany Medical College, Division of Nephrology and Hypertension, Albany, NY, USA.; 2 Albany Medical College, Division of Pulmonary Critical Care, Albany, NY, USA.; 3 Albany Medical College, Department of Pathology, Albany, NY, USA.; 4 Albany Medical College, Department of Neuroscience and Experimental Therapeutics, Albany, NY, USA.; 5 Jersey Shore University Medical Center, Seton Hall-Hackensack-Meridian School of Medicine, Neptune, NJ, USA.

**Keywords:** Anti-Neutrophil Cytoplasmic Antibody-Associated Vasculitis, Glomerulonephritis, Anti-Glomerular Basement Membrane Disease, Double Positive Disease, Vasculite Associada a Anticorpo Anticitoplasma de Neutrófilos, Glomerulonefrite, Doença Antimembrana Basal Glomerular, Doença Positiva Dupla

## Abstract

**Background and objectives:**

Anti-neutrophil cytoplasmic autoantibodies (ANCA) associated vasculitis is a small vessel vasculitis with insufficient epidemiologic estimates in the United States. We aimed to determine demographic and clinical features of ANCA associated vasculitis patients presenting to a large tertiary care referral center in Upstate New York. Design, setting, participants, and measurements: A retrospective analysis of cases with pauci-immune GN on renal biopsy and clinical diagnosis of ANCA vasculitis presenting over 11 years was conducted. Outcomes of interest were: demographics, ANCA antibody positivity, patient and renal survival, and regional trends.

**Results:**

986 biopsies were reviewed, 41 cases met the criteria for inclusion: 18 GPA, 19 MPA, and 4 double positive (anti-GBM disease plus ANCA vasculitis). Mean age at presentation was 52.4 years (SD 23.7), 23 (56%) were male and median creatinine was 2.6 mg/dL. The median patient follow up was 77 weeks (IQR 10 - 263 weeks), with a 3-month mortality rate of 5.7% and a 1-year estimated mortality rate of 12%. Thirteen patients required hemodialysis at the time of diagnosis; 7 patients came off dialysis, with median time to renal recovery of 4.86 weeks (IQR 1.57 - 23.85 weeks). C-ANCA positivity (*p* < 0.001) and C-ANCA plus PR3 antibody pairing (*p* = 0.005) was statistically significant in GPA *versus* MPA. P-ANCA positivity was observed in MPA *versus* GPA (*p* = 0.02) and double positive *versus* GPA (*p* = 0.002), with P-ANCA and MPO antibody pairing in MPA *versus* GPA (*p* = 0.044). Thirty-seven of the 41 cases were referred locally, 16 cases were from within a 15-mile radius of Albany, Schenectady, and Saratoga counties.

**Conclusions:**

ANCA vasculitis is associated with end stage renal disease and increased mortality. Our study suggests the possibility of higher regional incidence of pauci-immune GN in Upstate New York. Further studies should investigate the causes of clustering of cases to specific regions.

## RESUMO

**Introdução e objetivos:**

A vasculite associada a anticorpos anticitoplasma de neutrófilo (ANCA) é uma vasculite de pequenos vasos com estimativas epidemiológicas insuficientes nos Estados Unidos. Nosso objetivo foi determinar características demográficas e clínicas de pacientes com vasculite associada à ANCA, apresentando-se a um grande centro de referência de atendimento terciário em Upstate New York. Formato, cenário, participantes e medidas: Foi realizada uma análise retrospectiva dos casos de GN pauci-imune em biópsias renais e diagnóstico clínico de vasculite ANCA por mais de 11 anos. Os resultados de interesse foram: dados demográficos, positividade de anticorpos ANCA, sobrevidas renal e de pacientes e tendências regionais.

**Resultados:**

986 biópsias foram revisadas, 41 casos preencheram os critérios de inclusão: 18 GPA, 19 PAM, e 4 duplo-positivos (doença anti-MBG com vasculite ANCA). A média de idade na apresentação foi de 52,4 anos (DP 23,7), 23 (56%) eram do sexo masculino e mediana de creatinina de 2,6 mg/dL. O acompanhamento mediano dos pacientes foi de 77 semanas (IQR 10 - 263 semanas), com uma taxa de mortalidade de 3 meses de 5,7% e uma taxa de mortalidade estimada em 1 ano de 12%. Treze pacientes necessitaram de hemodiálise no momento do diagnóstico; 7 pacientes saíram da diálise, com tempo médio para recuperação renal de 4,86 semanas (IQR 1,57 - 23,85 semanas). A positividade para C-ANCA (*p* < 0,001) e o pareamento de anticorpos C-ANCA mais PR3 (*p* = 0,005) foram estatisticamente significantes em GPA *versus* PAM. A positividade de P-ANCA foi observada em PAM *versus* GPA (*p* = 0,02) e duplo positivo *versus* GPA (*p* = 0,002), com pareamento de anticorpos P-ANCA e MPO em PAM *versus* GPA (*p* = 0,044). Trinta e sete dos 41 casos foram encaminhados localmente, 16 casos foram de dentro de um raio de 15 milhas dos condados de Albany, Schenectady e Saratoga.

**Conclusões:**

A vasculite por ANCA está associada à doença renal terminal e aumento da mortalidade. Nosso estudo sugere a possibilidade de maior incidência regional de GN pauci-imune no norte do estado de Nova York. Novos estudos devem investigar as causas do acúmulo de casos em regiões específicas.

## INTRODUCTION

Anti-neutrophil cytoplasmic autoantibodies (ANCA) associated vasculitis (AAV) is a small vessel vasculitis associated with ANCA in 90% of cases^1^, and can manifest as 3 phenotypically distinct processes: granulomatosis with polyangiitis (GPA), microscopic polyangiitis (MPA), and eosinophilic granulomatosis with polyangiitis (EGPA). AAV presents with multi-organ involvement secondary to inflammation and necrosis within small blood vessels and can result in increased morbidity and mortality.

Population-based reports for AAV estimate an annual incidence of 13-21.8 per million.^2^^-^ 6 The incidence of GPA in the United Kingdom (UK) from the UK General Practice Research Database was reported at 8.4 per million until 2005 (295 cases of GPA from 3.6 million patients)^7^ and 11.8 per million person-years between 1997-2003 (462 cases of GPA)^8^ Watts *et al*. found a comparable incidence of MPA, i.e., 8.0 per million in a 15-year UK cohort followed until 1997.^9^ A 5-year prospective Japanese study reported a higher incidence of AAV (primarily MPA) of 22.6 per million per year.^10^ However, population based studies for incidence of AAV in the United States are lacking.

Our study was prompted by an appreciation of the increasing number of cases of AAV at Albany Medical College (AMC). We hypothesized an increased rate of renal biopsy-proven AAV amongst patients diagnosed at AMC and investigated the regional incidence of AAV.

## MATERIALS AND METHODS

Institutional review board (IRB) approval was obtained from the AMC IRB committee. Patients with pauci-immune necrotizing or crescentic GN on renal biopsy between January 1, 2005 to April 30, 2017, or with evidence of necrotizing GN and histopathological features suggestive of AAV (including those with a secondary diagnosis such as anti-glomerular basement membrane, i.e. anti-GBM disease, diabetes *mellitus*, hypertensive nephrosclerosis, and other secondary processes), and a confirmed clinical diagnosis of AAV, based upon the American College of Rheumatology (ACR) 1990^11^ classification criteria for GPA, and the revised Chapel Hill Consensus Criteria (CHCC) for MPA^12^, were included in the study. Outcomes of interest were: demographics, ANCA, anti-myeloperoxidase (anti-MPO), anti-proteinase 3 (anti-PR3), anti-glomerular basement membrane (anti-GBM) antibody positivity, patient and renal survival, and regional trends. Patients were excluded if the renal biopsy did not list AAV as a differential diagnosis. We excluded patients who had pauci-immune GN on renal biopsy, but an inconsistent clinical diagnosis, i.e. another process that was more likely than AAV. Additionally, patients with inadequate clinical data at the time of renal biopsy, with absence of more than 50% of laboratory test results or clinical parameters were excluded.

Degree of proteinuria was determined using the 24-hour urine protein, and if unavailable, the spot urine protein and creatinine ratio was utilized.

Histopathological class was determined by our pathologist using the original biopsy reports to classify patients according to the 2010 Berden histopathological classification for AAV.^13^ This classification system is not applicable to patients with a secondary diagnosis on renal biopsy, hence we did not designate a histopathological class to such cases. The need for dialysis therapy at the time of presentation was assessed. In addition, the number of patients coming off dialysis as well as remaining on long-term dialysis therapy was also evaluated (renal outcome).

Comparisons of normally distributed continuous data were done by independent sample *t*-tests when comparing two groups or by analysis of variance with Tukey's test for multiple comparisons when comparing three or more groups. Data were summarized by the mean and standard deviation. For data that displayed marked departures from normality, we used Mann Whitney tests for two groups or Kruskal Wallis tests for more than two groups. These data are summarized by the median and the interquartile ranges (IQR, 25^th^ and 75^th^ percentile). Comparison of categorical data was done by chi-squared tests (Fisher's exact test if expected values were less than 5). Linear regression was used to test relationships between continuous variables. Kaplan-Meier curves were used for assessing time to event data with the log rank test applied for testing between groups. Minitab statistical software was used for all data analysis with significance accepted at *p* < 0.05.

## RESULTS

**Figure 1:**
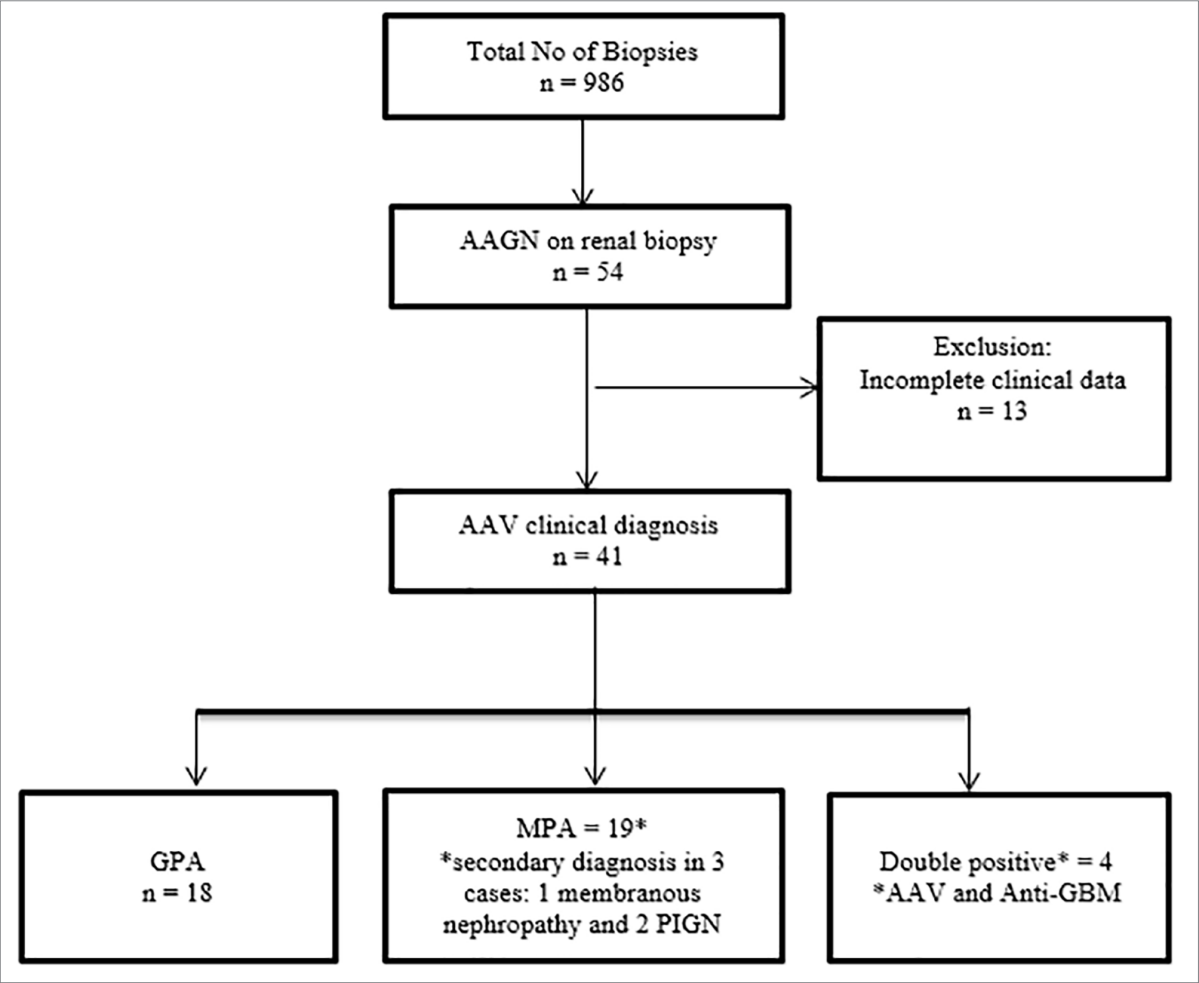
Flowchart for ANCA associated vasculitis (AAV) case selection.

Nine hundred and eighty-six native kidney biopsies were reviewed, and 54 met criteria for histopathological diagnosis of AAV, i.e. consistent with pauci-immune GN (Figure 1). Of these, 13 cases had incomplete initial clinical data. Hence, 41 cases were included in this analysis.

Demographic characteristics are presented in Table 1. Thirty-seven (90%) of the 41 patients were Caucasians (based on self-reported ethnicity) and 23 (56%) were males. The age of presentation was 52.4 years (SD 23.7). There were 18 GPA, 19 MPA, and 4 double positive disease (anti-GBM antibody and ANCA positive). There was no statistically significant difference in gender amongst the 3 groups, i.e. GPA, MPA, and double positives (*p* = 0.18). The median creatinine at the time of biopsy was 2.6 mg/dL (IQR 1.5-4.9), with highest median serum creatinine of 6.9 mg/dL (IQR 1.4 - 14.2) in double positive disease, *versus* 3.3 mg/dL (IQR 1.5-5.1) in GPA, and 2.4 mg/dL (IQR 1.5 to 4.3) in MPA (Table 2). Mean serum hemoglobin was 9.7 (SD 1.8) gm/dL, with no statistically significant difference between GPA, MPA, and double positives (*p* = 0.74). Mean serum albumin was 2.72 (SD 0.68) gm/dL with nephrotic range proteinuria in 16% of the cases, and microscopic hematuria in all cases (Table 1). Three cases in the MPA group had a secondary diagnosis (1 membranous nephropathy and 2 post-infectious GN i.e. PIGN); however, only the patient with membranous nephropathy had nephrotic range proteinuria.

**Table 1:** Baseline Characteristics

	Proportion	Mean or Median (SD or IQR)
Age		52.41 (23.71)
Gender		
Males		
	23/41 (56%)	
Race		
White	37/41 (90%)	
African American	1/41 (2.4%)	
Asian	1/41 (2.4%)	
Hispanic	1/41 (2.4%)	
American Indian	1/41 (2.4%)	
		
Hypertension	30/41 (73%)	
Albumin		2.72 gm/dL (SD 0.68)
Hemoglobin		9.69 gm/dL (SD 1.81)
Proteinuria		2.28 gm (SD 2.2)
Proteinuria > 3.5 gm	6/36 (16%)	
Hematuria	41/41 (100%)	
		
Baseline Creatinine (n = 28)		1.18 mg/dL (0.43)
Creatinine at time of biopsy		2.6 mg/dL (IQR 1.5-4.9)
GPA (18)		3.25 mg/dL (IQR 1.48-3.25)
MPA (19)		2.4 mg/dL (IQR 1.5-4.3)
Double positive (4)		6.9 mg/dL (IQR 1.35-14.17
		
Dialysis		
All	13/41 (32%)	
GPA (18)	6/18 (33%)	
MPA (19)	5/19 (26%)	
Double positive (4)	2/4 (50%)	
		
C-ANCA (40)	23/40 (58%)	
P-ANCA (41)	14/41 (34%)	
MPO (37)	12/37 (32%)	
PR-3 (38)	17/38 (45%)	
Atypical ANCA	1/37 (2.7%)	

**Table 2:** Baseline laboratory data and serology comparison by clinical diagnosis

Total N = 41	GPA (SD*)	MPA (SD)	Double positive (SD)	*p* value
Creatinine (41)	4.21 (4.12)	2.81 (1.55)	7.47 (6.86)	0.062
Albumin (41)	2.62 (0.62)	2.69 (0.72)	3.3 (0.64)	0.197
Hemoglobin (41)	9.75 (1.66)	9.50 (1.79)	10.28 (2.95)	0.74
C-ANCA (40)	16/17	7/19	0/4	< 0.001
P-ANCA** (41)	1/18	9/19	4/4	< 0.001

* SD = standard deviation

** Double positive versus GPA (*p* = 0.002), MPA versus GPA (*p* = 0.02), Double positive versus MPA (*p* = 0.28) with Fisher’s exact test using Bonferroni correction

## PATIENT SURVIVAL

**Figure 2:**
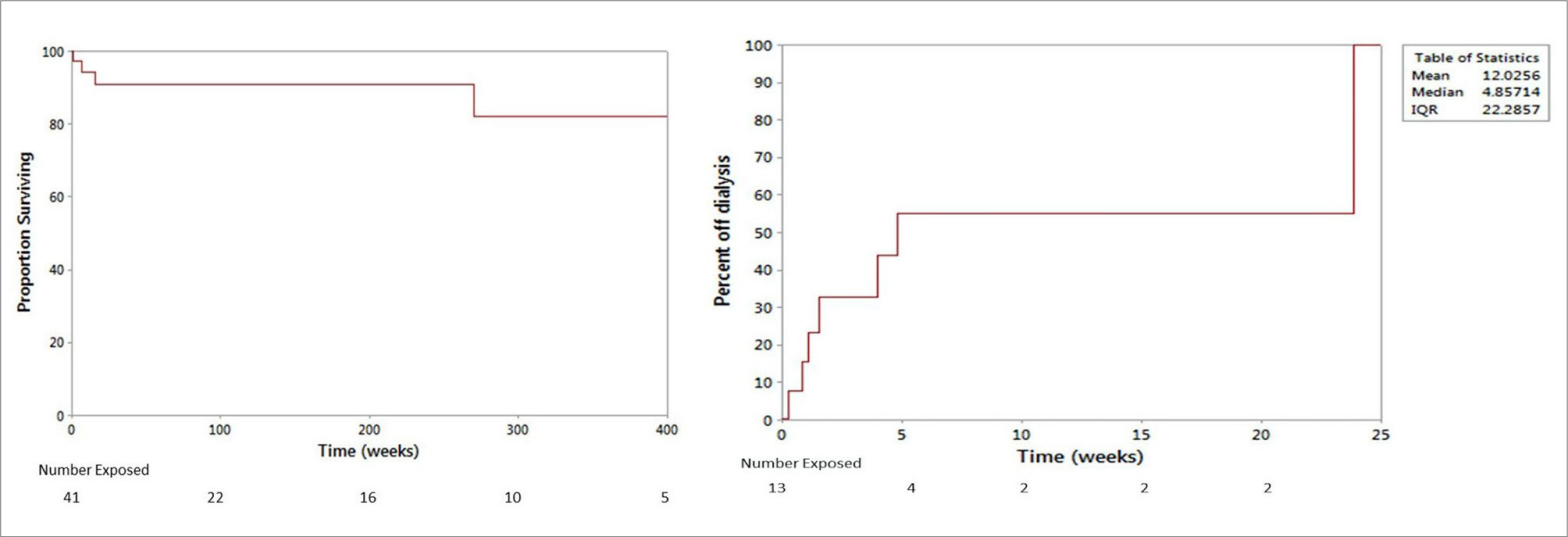
Kaplan-Meier survival curves describing (a) patient survival (b) renal outcomes: proportion off dialysis. Patient with the longest follow-up came off hemodialysis at the end of 23 weeks.

The median patient follow up was 77 weeks (IQR 10 - 263 weeks) (Figure 2a). There were 4 deaths amongst the 41 cases, 3 patient deaths were within 16 weeks of diagnosis of AAV, and 1 death at 5.2 years. Three patients were dialysis-dependent at the time of death. The 3-month mortality rate was 5.7%, and the 1-year estimated mortality rate was 12%, with 12-month follow up data available for 22 patients (3 deaths during this time period).

## RENAL OUTCOMES: DIALYSIS FREE SURVIVAL

Thirteen patients required hemodialysis at the time of diagnosis of AAV. Seven of these patients came off dialysis (6 treated with cyclophosphamide-based regimen, 1 received rituximab); the patient with the longest follow-up came off hemodialysis at the end of 23 weeks (Figure 2b). Six patients who remained on dialysis (4 treated with cyclophosphamide-based regimen, 1 with rituximab, and 1 did not receive immediate immunosuppression due to bacteremia) were transferred to dialysis units outside AMC and were documented to be receiving dialysis therapy at the time of last follow-up.

For patients that came off hemodialysis, median time to renal recovery was 4.86 weeks (IQR 1.57 to 23.85 weeks). There was no significant difference in serum creatinine (*p* = 0.54), hemoglobin (*p* = 0.14), or albumin (*p* = 0.62) amongst patients who remained on dialysis, *versus* those who demonstrated renal recovery and came off dialysis.

## ANTIBODY POSITIVITY

C-ANCA was positive in 16 of the 17 cases with GPA *versus* 7 of the 19 cases with MPA, and none of the double positive cases (*p* < 0.001). One case with GPA had missing C-ANCA result. P-ANCA antibody was positive in 9 of 19 MPA, all 4 double positive cases, and only 1 of 18 GPA (*p* < 0.001). Statistical differences within groups for P-ANCA positivity were confirmed in double positive *versus* GPA (*p* = 0.002), and MPA *versus* GPA (*p* = 0.02), but not in double positive *versus* MPA (*p* = 0.28, Fisher's exact test using Bonferroni correction) (Table 2). Three patients were ANCA negative (by indirect immunofluorescence and ELISA testing for MPO and PR3), despite renal biopsies consistent with pauci-immune GN and clinical diagnosis of AAV.

## C-ANCA AND PR3 PAIRING/P-ANCA AND MPO PAIRING

C-ANCA and PR3 antibody pairing was present in 11 of 16 GPA, *versus* 3 of 17 MPA (*p* = 0.005). Two cases in each group had missing results for either C-ANCA or PR3 antibodies. P-ANCA and MPO antibody pairing was confirmed in 5 of 17 MPA and none of the GPA cases (*p* = 0.044). Three of 4 double positives had p-ANCA and MPO pairing, while 1 had p-ANCA and PR3 pairing.

## HISTOPATHOLOGICAL CLASSIFICATION

Renal biopsy reports of 34 cases with GPA or MPA were reviewed by our pathologist to assign a histopathological category. The most common histopathological class was focal (16 cases), followed by crescentic (11 cases), mixed (5 cases), and the least common class was sclerotic (2 cases). Seven cases had a secondary diagnosis (4 double positives, and 3 cases with MPA) and thus were not assigned a histopathological class.

## ZIP CODE MAPPING

**Figure 3:**
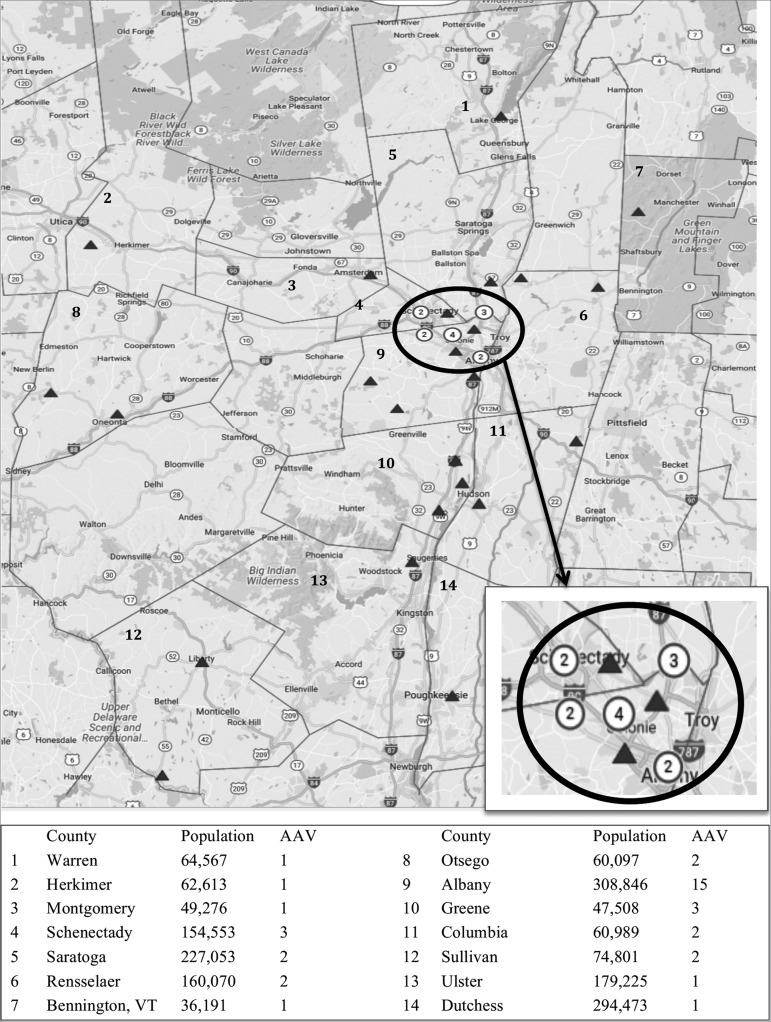
Regional mapping of ANCA associated vasculitis (AAV) cases by county. Zone of increased frequency of AAV cases magnified bottom right, highlighting 16 cases in a 15-mile radius between Albany, Schenectady and Saratoga County. Population estimates from U.S. Census Bureau.^14^

Cases were mapped to zip codes on file (Figure 3). Four cases were from distant states, while thirty-seven cases were locally referred from nearby counties; sixteen of these resided within a fifteen-mile radius of Albany, Schenectady, and Saratoga counties.

## TEMPORAL TRENDS

**Figure 4:**
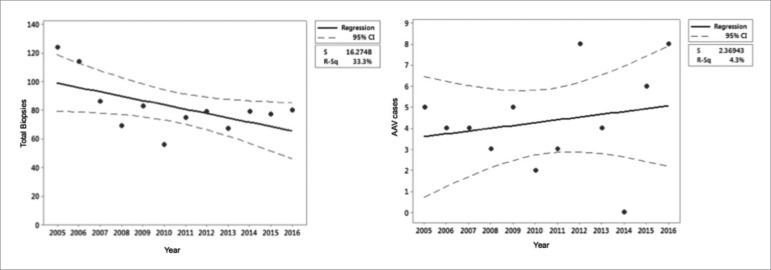
Renal biopsies at Albany Medical Center from January 1, 2005 to December 31, 2016 illustrating (a) total biopsies (b) ANCA associated vasculitis (AAV) biopsies.

We did not include cases from the first quarter of 2017 for the analysis of trends in the incidence of pauci-immune GN amongst kidney biopsies at AMC. While there was no statistically significant increase in the number of confirmed AAV cases during this time period, there was a trend towards decreased total number of biopsies (*p* = 0.05) from 2005 to 2016 (Figure 4). Of note, 8 of the 41 cases presented in 2016.

## DISCUSSION

While population-based incidences for AAV in the United States (U.S.) are limited, data on regional trends is emerging. Pauci-immune GN was reported in 7.9% of 21,794 kidney biopsies over 30 years at the University of North Carolina (UNC) in southeastern US,^15^ which is higher than the percentage of confirmed AAV (5.4%) at our center. Notably, we did not have clinical data for 13 patients excluded from the study despite presenting pauci-immune GN on renal biopsy. Accounting for these patients, the percentage of pauci-immune GN at our center is similar to that reported by UNC.^15^ Additionally, UNC is a large referral center for renal biopsies, and the reported percentages may represent a greater proportion of referred biopsies, rather than a true estimate of local disease trends. In comparison, most renal biopsies at AMC are from AMC Hospital, with a small proportion from Albany Stratton Veterans Administration Medical Center (VAMC), and local nephrology groups.

AAV has a higher incidence in the 55-69-year-old group,^8^ although it has been reported across all ages. The mean age at the time of diagnosis in our cohort was 52.4 (SD 23.7), and 56% were male. The male to female ratio for GPA in the 1990 ACR Classification was 1.7:1.^11^, and this marginal male predominance has been reported in other studies previously.^8^^,^ 16

AAV is more commonly seen in Whites (93 - 98% of patients)^17^ although MPA is well reported among Asians in Japan and China.^10^^,^ 18 ^,^ 19 AAV is rare in AA, and genotyping of MHC class II alleles by Cao *et al*. revealed that AA patients with PR3-ANCA had a higher incidence of HLA-DRB1*15 alleles than controls in the community (OR 73.3; 95% CI 9.1 to 591).^20^ The association with DRB1*15 allele was also noted among Caucasian patients with PR3-ANCA (OR 2.2; 95% CI 1.2 to 4).^20^

ANCA screening is performed by indirect immunofluorescence microscopy on neutrophils to detect predominantly central (C-ANCA) or cytoplasmic (P-ANCA) staining. Antibodies against myeloperoxidase (MPO) and proteinase 3 (PR3) antigens expressed on the surface of neutrophils and monocytes are recognized as the main ANCA targets.^1^^,^ 21 The antigen specific ELISA testing for anti-MPO and anti-PR3, in addition to ANCA indirect immunofluorescence, increases the sensitivity of the results to 90% and specificity to 70% for the diagnosis of AAV.^22^

PR3-ANCA positivity with cytoplasmic pattern on immunofluorescence, i.e. C-ANCA and PR3 pairing, is seen in 90% patients with GPA,^17^^,^ 23 while P-ANCA and MPO antibodies result positive in 60-80% of patients with MPA.^16^ EGPA has the lowest proportion of ANCA positivity (25% in patients without renal involvement, 75% in patients with renal involvement, and 30-40% of all cases are seropositive for ANCA), and MPO pairing with P-ANCA is most commonly seen.^17^^,^ 24 Our results were consistent with these observations, with C-ANCA (*p* < 0.001) and C-ANCA with PR-3 pairing (*p* = 0.005) in GPA; P-ANCA (*p* = 0.02), and P-ANCA with MPO (*p* = 0.044) pairing in MPA.

In a recent review on anti-GBM disease, McAdoo *et al*. conclude that anti-GBM positivity is reported in 5-10% of AAV.^25^^-^ 28 The proportion of double positive disease in our study was comparable, with 4 of 41 (approximately 10%) cases of AAV testing positive for anti-GBM antibody at the time of renal biopsy. While double positives demonstrate disease severity typical of anti-GBM disease on initial presentation, anti-GBM diseases have a low relapse rate, and relapse in double positive cases is primarily driven by AAV.^29^ An estimated 50% of patients with double positive disease are expected to relapse.^29^

In a meta-analysis of 44 studies on clinical outcomes of AAV by Mukhtyar *et al*., 5-year survival for GPA, MPA, and EGPA were estimated at 74-91, 45-76, and 60-97%, respectively.^30^ Mortality rates for GPA were similar in a large UK cohort, and reported at 30 days, 90 days, and 1 year as 4.8, 9.9 and 13.6%.^8^ The 3-month and 1-year mortality rates in our cohort were 5.7% and 12%. These findings are comparable to the prior studies reported on this subject. The mortality rates in our study may be influenced by lack of follow-up.

Lee *et al*. performed a retrospective study on predictors of treatment outcomes in 155 patients with AAV. In this study, 87% of patients with AAV required dialysis on presentation; 51% responded to immunosuppression and came off dialysis at 4.4 weeks (range 1-39 weeks).^31^ Out of the remaining 49%, 14% died and from the 35% that were dialysis-dependent at 4 months, an additional 3 patients recovered renal function by the end of the first year after diagnosis.^31^ Koldingsnes *et al*. reported renal recovery 3 months after diagnosis of AAV in 4 of 8 patients that required hemodialysis at presentation, with an additional 3 patients temporarily coming off dialysis for 32 months (range 10-91 months) before progressing to ESRD.^32^ Our findings were similar, with 53.8% (seven) patients regaining renal function sufficient to come off dialysis by the end of week 23.

Berden *et al*. proposed a histopathological classification for AAV with four classes: focal, mixed, crescentic, and sclerotic, and correlated these to clinical outcomes.^13^ Renal survival was most favorable for focal, then crescentic, mixed, and sclerotic at 1 and 5-year follow-up.^13^ Bjørneklett *et al*. used this classification to review 250 cases with ANCA-associated GN, and reported a trend towards lowest risk for ESRD at 1 and 5 years in the focal class, followed by mixed and crescentic, and least favorable renal outcomes in sclerotic.^33^ However, a multivariate analysis accounting for GFR at the time of biopsy did not confirm a significant difference for renal outcomes between the classes.^33^ The prevalence of histopathological classes in our cohort differed from the validation study for the Berden classification, in which the most common class was crescentic. The classes in our cohort in decreasing frequency were: focal, crescentic, mixed, and sclerotic.^13^ Due to variable follow-up time and relatively smaller number of cases in each class, analysis for renal outcomes was not performed.

Environmental triggers for AAV as reviewed by Morgan *et al*.^34^ and prior studies, have included silica, farming exposure (more with livestock than crops), and solvents.^18^^,^ 35 ^-^ 37 Hogan *et al*. performed a case-control study using patient reported data and found increased exposure to silica dust in patients with AAV compared to controls (*p* = 0.001).^36^ Increased regional frequency and severity of pulmonary symptoms in MPO-AAV was reported after the earthquake in Kobe, Japan^18^, although a similar increase in incidence or change in clinical manifestations was not appreciated after the Christchurch earthquake in New Zealand.^16^ Possibilities considered for the variable findings after the earthquakes were differences in air quality, dust pollution, earthquake damage, and construction material.^16^ These differences in exposure to region-specific triggers may explain varying local incidences and spatial trends.

To further explore regional trends, we mapped the 41 cases in our study. Thirty-seven of the 41 cases were referred locally, and there was a cluster of 16 patients within a 15-mile radius among the three counties. Our findings suggest the possibility of higher regional incidence of pauci-immune GN. These results likely represent the lower limit of regional estimates, as our study was limited to cases presenting to AMC with renal involvement. Population-based studies with local and national registries are needed to determine the true incidence of this disease.

While there was no statistically significant change in temporal trends of confirmed AAV among kidney biopsies at AMC, 13 cases with biopsies consistent with pauci-immune GN were excluded from the study, and may have had a substantial impact on these outcomes. Of note, 8 of the 41 cases of AAV presented in 2016. The total number of renal biopsies from 2005 to 2016 also decreased (*p* = 0.05) due to change in local biopsy referral patterns, as the Albany Stratton VAMC and local private nephrology groups no longer refer biopsies to AMC.

Our data suggests that AAV primarily affects Caucasians and presents later in life, with mean age at presentation of 52.4 years in our cohort, with a marginal male predominance. Additionally, there appears to be regional variation in the number of AAV cases, with possible implicative factors including environmental and genetic factors. Antibody pairing for C-ANCA and PR-3 (*p* = 0.005) in GPA, P-ANCA with MPO (*p* = 0.044) in MPA, and 10% double positive cases (ANCA plus anti-GBM) was consistent with previously reported data. The 1-year mortality rate was estimated at 12%, with a third of all patients requiring dialysis on admission, and half of these cases (53.8%) demonstrating renal recovery by 23 weeks.

Our study has a referral bias, with more severe cases of AAV transferred to AMC, as it is a large tertiary care center. The outcomes and mortality estimates may be skewed by these factors, with less severe cases of AAV not being referred to AMC, and treated at local hospitals. Additionally, the use of renal biopsy to identify cases of RPGN may lead to a selection bias for cases with increased severity of illness.
